# The Impact of Impulsivity on Weight Loss Four Years after Bariatric Surgery

**DOI:** 10.3390/nu8110721

**Published:** 2016-11-14

**Authors:** Kathrin Schag, Isabelle Mack, Katrin E. Giel, Sabrina Ölschläger, Eva-Maria Skoda, Maximilian von Feilitzsch, Stephan Zipfel, Martin Teufel

**Affiliations:** 1 Medical Hospital, Department of Psychosomatic Medicine and Psychotherapy, University of Tübingen, 72076 Tübingen, Germany; kathrin.schag@med.uni-tuebingen.de (K.S.); katrin.giel@med.uni-tuebingen.de (K.E.G.); sabrina.oelschlaeger@student.uni-tuebingen.de (S.Ö.); eva-maria.skoda@med.uni-tuebingen.de (E.-M.S.); stephan.zipfel@med.uni-tuebingen.de (S.Z.); martin.teufel@med.uni-tuebingen.de (M.T.); 2 Department of General, Visceral and Transplant Surgery, University of Tübingen, 72076 Tübingen, Germany; maximilian.feilitzsch@med.uni-tuebingen.de

**Keywords:** weight loss, bariatric surgery, impulsivity, depression, eating behavior

## Abstract

Bariatric surgery has serious implications on metabolic health. The reasons for a failure of bariatric surgery, i.e., limited weight loss, are multifactorial and include psychological factors. We established a theoretical model of how *impulsivity* is related to weight loss outcome. We propose that depressive symptoms act as a mediator between impulsivity and *pathological eating behavior*, and that pathological eating behavior has a direct impact on weight loss outcome. We calculated *excessive weight loss* (%EWL) and assessed self-reported impulsivity (using the Baratt Impulsiveness Scale (BIS-15) total score), depressive symptoms (the Patient Health Questionnaire (PHQ-9) score), and pathological eating behavior (the Eating Disorder Inventory 2 (EDI-2) total score) in 65 patients four years after laparoscopic sleeve gastrectomy. Regression and mediation analyses were computed to validate the theoretical model. The BIS-15, PHQ-9, and EDI-2 have medium to high correlations between each other, and EDI-2 correlated with %EWL. The mediation analysis yielded that the PHQ-9 represents a significant mediator between BIS-15 and EDI-2. The regression model between EDI-2 and %EWL was also significant. These results support our theoretical model, i.e., suggest that impulsivity has an indirect impact on weight loss outcome after bariatric surgery, mediated by depression and transferred through pathological eating behavior. Thus, the underlying psychological factors should be addressed in post-operative care to optimize weight loss outcome.

## 1. Introduction

Bariatric surgery is the most effective treatment for patients with severe obesity that on average results in 20%–35% of body weight loss from initial weight after 2–3 years [1] and substantially decreases somatic comorbidities associated with metabolic syndrome [1,2,3]. About one fifth of patients do not achieve 50% *excess weight loss* (EWL) [4,5], which is the common threshold for successful weight loss after bariatric surgery. The predictors for a failure of bariatric surgery are multifaceted reaching from surgical complications to intrapersonal factors such as psychopathology, *pathological eating behavior*, and personality traits [5,6,7,8,9,10,11].

*Impulsivity* might be one personality factor contributing to a limited benefit from bariatric surgery [12]. Impulsivity is considered to be a fairly stable personality trait. It is a multifactorial trait with a neurobiological basis in the mesolimbic reward system and the prefrontal cortex [13,14,15]. It leads to rash and spontaneous behavior without consideration of possible consequences (i.e., the impulsivity component “disinhibition”), especially in highly positive mood (i.e., the “extraversion/sensation seeking” component) or highly negative mood (i.e., the “neuroticism/negative urgency” component) [14,15].

Current reviews deliver evidence that patients with obesity and especially those who seek bariatric surgery show increased impulsivity and related neurological impairments [12,16,17]. On a behavioral level, impulsive people tend to eat more unhealthy foods [18], and there is evidence that impulsivity in patients with obesity is especially emerging in terms of *pathological eating behavior* such as binge eating or food addiction [16,17,19]. Moreover, pathological eating is increased in patients with obesity seeking bariatric surgery [4,20]. Particularly, the diagnosis of a binge eating disorder (BED) in patients who wait for bariatric surgery, with a point prevalence of 13.4% and 14.6% of subthreshold binge eating, is remarkably high [21], compared with the lifetime prevalence of 1.1%–1.9% in the general population [22]. Concerning the impact of the *affective state*, several studies indicate that people with obesity show pathological eating behavior especially in negative mood [23], which is in line with the negative urgency concept of impulsivity [15]. Likewise, a high rate of patients seeking bariatric surgery suffers from depressive symptoms, and depression is the most common mental disorder in this patient group [4,8]. In sum, impulsivity, depression, and pathological eating behavior are closely connected to each other. For example, Pearson and colleagues [24] showed that both impulsivity (negative urgency) and depression predict pathological eating behavior.

Concerning the way in which psychological factors such as impulsivity influence *EWL* in patients with bariatric surgery, evidence is insufficient and mixed [1,7], especially concerning post-operative factors [25]: Konttinen and colleagues [26] showed that less post-operative impulsivity and the amount of decrease in impulsivity after surgery is associated with a higher amount of weight loss at a 10-year follow-up. However, some researchers [12,27] did not find a predictive value of impulsivity on EWL after bariatric surgery. Thus, Gerlach and colleagues [12] propose that pathological eating behavior might mediate the association between impulsivity and weight loss outcome. This hypothesis is in line with studies that found that post-operative pathological eating behavior, especially uncontrolled eating, predicts decreased EWL after bariatric surgery [25,28]. For example, we showed in a recent study [29] that post-operative eating behavior measured with the Eating Disorder Examination explains 36% of the variance of EWL. Concerning depression, post-operative depressive symptoms are associated with decreased EWL after bariatric surgery, but the causal direction is unknown [25,30]. In the general population, the same association was found [26], and Van Strien and colleagues [31] showed that pathological eating is a mediator between depression and weight gain.

**Figure 1:**
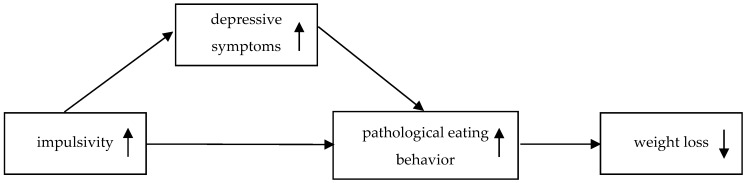
Theoretical model of the relation between impulsivity and weight loss outcome after bariatric surgery.

Taking together, we developed a theoretical model of how impulsivity could be related to weight loss outcome in patients with obesity after bariatric surgery (Figure 1). In this model, we hypothesize that impulsivity is associated with pathological eating behavior and that this association is mediated by depression according to the negative urgency component of impulsivity [15]. In contrast to Pearson and colleagues [24] who used the negative urgency scale to measure impulsive behavior, we are convinced that it is more useful to assess impulsivity separately from depression to disentangle both concepts. Further, we hypothesize in accordance with Mack and colleagues [29] that pathological eating behavior as the most behavioral factor is directly associated with weight loss (Figure 1). To investigate if this model holds true, we explored EWL and the self-reported psychological outcomes in the sample from Mack and colleagues [29] four years after laparoscopic sleeve gastrectomy.

## 2. Materials and Methods

This study is part of the project from Mack and colleagues [29]. It was approved in March 2013 by the Ethics Committee of the University Hospital Tübingen, Germany (No.: 727/2012BO2) and is registered at the German Clinical Trials Register (No.: DRKS00005130 available at https://drks-neu.uniklinik-freiburg.de).

### 2.1. Participants and Procedure

For the present study, we assessed 65 individuals with severe obesity who received laparoscopic sleeve gastrectomy (LSG) 4 years (48 ± 14 months) before at the Comprehensive Obesity Centre of the University Hospital Tübingen, Germany. In 57 participants, body weight and height as well as the self-report questionnaires were assessed at a personal appointment. The remaining 8 participants completed the questionnaires by mail and gave self-report information about weight and height (*n* = 6), validated weight and height by a physician (*n* = 1), or did not report weight or height (*n* = 1).

### 2.2. Measurements

#### 2.2.1. Body Mass Index (BMI), Body Weight Loss (%BWL), and Excess Weight Loss (%EWL)

BMI 4 years after LSG was calculated according to body weight and height (kg/m^2^). On this basis, the %BWL and %EWL were calculated:

%BWL = 100 × (operative weight (kg) − post-operative weight (kg)/operative weight (kg)),(1)

and

%EWL = 100 × (operative weight (kg) − post-operative weight (kg))/overweight at operation relative to the ideal weight (kg)),(2)

where the ideal weight is defined as a BMI of 25 kg/m^2^. 

#### 2.2.2. Impulsivity

The German short version of the Baratt Impulsiveness Scale (BIS) [15,32] was used to assess impulsivity. The BIS-15 is a self-report questionnaire consisting of 15 items in 3 subscales, which are rated on a 4-point response format ranging from 1 = “rarely/never” to 4 = “almost always”. The BIS-15 total score ranges from 15 to 60. As we aimed to measure general impulsivity, we used the BIS-15 total score in the statistical analyses.

#### 2.2.3. Pathological Eating Behavior

The German version of the Eating Disorder Inventory 2 (EDI-2) [33] was used to assess eating-disordered attitudes and behaviors. The questionnaire consists of 91 items in 11 subscales with a 6-point response format ranging from 1 = “never” to 6 = “always”. The EDI-2 total score ranges from 91 to 546. As we aimed to measure general pathological eating behavior, we used the EDI-2 total score.

#### 2.2.4. Depression

The Patient Health Questionnaire, German version (PHQ-D) [34], is a screening tool for mental disorders. The 9-item module depression (PHQ-9) [35] determines the severity of depression. It has a 4-point response format ranging from 0 = “not at all” to 3 = “nearly every day”, resulting in a 0 to 27 severity score and a cut-off score of 5 for a mild depression and 10 for a moderate depression.

### 2.3. Statistical Analyses

The data were analysed with SPSS version 23 [36]. For patients with more than 20% missing items in the BIS-15 total score, the EDI-2 total score or the PHQ-9 score, data was excluded. For patients below 20% missings per scale, missing data were completed using the Expectation–Maximization Algorithm [37].

The data were tested for normal distribution using the Kolmogorov-Smirnov test. Pearson correlations were conducted if the data were parametric, and Spearman correlations if they were non-parametric. If the variables correlated as expected in our theoretical model, we computed regression and mediation analyses to test the theoretical model and determine the amount of explained variance. In these analyses, the term “determinants” is used instead of “predictor”, because we assessed variables cross-sectionally. In spite of cross-sectional data, we found it reasonable to compute mediation analyses, because impulsivity as a personality trait is expected to stay stable over time; thus, it is assumed to be the foregoing factor in our theoretical model (Figure 1). Concerning the mediation analysis, we used the PROCESS macro for SPSS and SAS version 2.16 [38] and computed a bootstrapping analysis with 10.000 samples and 95% confidence interval according to Preacher and Hayes [39]. A *p*-value of <0.05 was considered statistically significant. 

## 3. Results

### 3.1. Sample Characterisitics

Sample characteristics are presented in Table 1.

### 3.2. Correlational Analyses

We found several correlations between the examined variables (Table 2). The three psychological variables BIS-15 total score, EDI-2 total score, and PHQ-9 score correlated amongst each other with medium to high correlations, whereas %EWL and %BWL correlated significantly with the EDI-2 total score, but not with the BIS-15 total score or the PHQ-9 score. As %EWL and %BWL correlated highly with *r* = 0.94, *p* < 0.001, we decided to include only %EWL in further analyses.

### 3.3. Regression and Mediation Analyses

**Figure 2:**
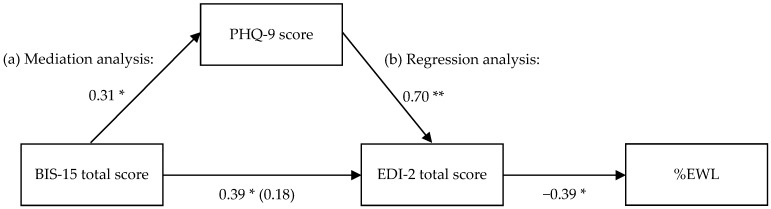
Mediation and regression analyses concerning the relationship between impulsivity, depression, pathological eating behavior, and weight loss: (a) Standardized regression coefficients for the relationship between the BIS-15 total score, the PHQ-9 score, and the EDI-2 total score. The standardized regression coefficient between the BIS-15 total score and the EDI-2 total score, controlling for the PHQ-9 score, is in parantheses. *n* = 50; (b) Standardized regression coefficient of the relationship between the EDI-2 total score and %EWL. *n* = 53. * *p* < 0.05, ** *p* < 0.001, two-sided.

We first computed a mediation analysis with the BIS-15 total score as determinant, the PHQ-9 score as mediator, and the EDI-2 total score as outcome. As Figure 2 illustrates, the standardized regression coefficient between BIS-15 total score and PHQ-9 score was significant as well as the regression coefficient between the PHQ-9 score and the EDI-2 total score. The simple regression with the BIS-15 total score as determinant and the PHQ-9 score as outcome yielded an explained variance of *R*^2^ = 9.4% (*F*(1,48) = 5.00, *p* = 0.030), and the simple regression with the PHQ-9 score as determinant and the EDI-2 total score as outcome yielded an explained variance of *R*^2^ = 56.6% (*F*(1,48) = 62.64, *p* < 0.001). The bootstrapped indirect effect IE of the BIS-15 total score on the EDI-2 total score through the PHQ-9 score was statistically significant with CI [0.14, 4.05] (unstandardized IE = 1.93; standardized IE = 0.21). The direct effect between the BIS-15 total score and the EDI-2 total score was not significant, if we controlled for the PHQ-9 score (*p* = 0.077, Figure 2). Thus, the association between the BIS-15 total score and the EDI-2 total score was mediated by the PHQ-9 score.

Finally, we computed a simple regression analysis between the EDI-2 total score as determinant and %EWL as outcome. The regression model was significant (*F*(1, 51) = 9.3, *p* = 0.004) with an explained variance of *R*^2^ = 15.4%. The standardized regression coefficient is presented in Figure 2.

## 4. Discussion

The validation of theoretical models can help to elucidate the factors contributing to limited weight loss after bariatric surgery. This is the first study that tested a model concerning the impact of post-operative impulsivity on weight loss outcome in bariatric surgery patients in a four-year follow-up. Here, we suggest an indirect relationship between impulsivity and weight loss (Figure 1). Our results suggest that the relationship between impulsivity and pathological eating behavior is mediated by depressive symptoms, and that pathological eating behavior in turn has a direct effect on weight loss (Figure 2).

As expected, impulsivity and depressive symptoms did not correlate with weight loss, but both are associated with pathological eating behavior—depressive symptoms especially correlated highly. Hence, the relationship between impulsivity and pathological eating behavior was mediated by depressive symptoms. It might be that this is due to the concept of “negative urgency” [15], the tendency to act impulsively, especially in negative mood. The high amount of explained variance (56.6%) from the pathological eating behavior by depressive symptoms suggests a close link between these two concepts. The obviously lower, but still substantial, amount of explained variance from impulsivity on depressive symptoms (9.4%) speaks for a multifactorial solution. 

The significant regression between post-operative pathological eating behavior and weight loss is in line with other studies [25,28] such as our study from Mack and colleagues [29]. These studies indicate that pathological eating behavior persists in a substantial percentage of bariatric surgery patients and that these habits contribute to limited weight loss. According to our current results, at least 15.4% of weight loss outcome were explained by self-reported pathological eating behavior, showing that this has a relevant impact and should be addressed in follow-up care.

Taken together, our results are partly in line with the hypothesis that pathological eating behavior mediates the association between weight and impulsivity [12] or between weight and depression [31]. We also identified a link between these four variables, but as weight loss did not correlate with impulsivity and depression, pathological eating behavior could not serve as a mediator between those variables. Our results fit also partly to Pearson and colleagues [24] who found two simultaneous pathways of impulsivity and depression predicting binge eating behavior in children. In bariatric surgery patients, impulsivity and depression seem to be more intertwined in predicting pathological eating behavior. Compared with these three meaningful studies, we go one step further, understanding depression as a mediator between impulsivity and pathological eating behavior, which in turn impacts weight loss. 

Deducing a clinical view from these results, a bariatric surgery patient with an impulsive personality and depressive symptoms shows a high degree of pathological eating behavior, which in turn limits weight loss. Thus, it is important to address these factors lying beyond pathological eating behavior to achieve sustainable long-term changes in eating behavior and therefore weight loss success and weight loss maintenance.

In a field of missing knowledge, this study provides first insights into relevant underlying psychological mechansims of limited weight loss after bariatric surgery by validating an a priori generated theoretical model with well-thought-out statistical methods. Nonetheless, it is important to note some of the limitations of this project: Overall, our suggested model needs to be replicated in an independent sample with longitudinal data. As we assessed psychological outcomes and weight loss cross-sectionally, we cannot conclude any causal relationships. This represents a clear limitation of this study. Further, using cross-sectional data in mediation analyses could lead to biased results [40]. Therefore, it would be important to explore post-operative psychological variables at a preceding time point of weight loss. Furthermore, observing an alteration of these variables after a post-operative intervention program, could give more insight. Wild and colleagues [41] indicated that a videoconferencing-based psychoeducational group following bariatric surgery could reduce depressive symptoms. To reduce costs and patient efforts to participate in such a group program, stepped care interventions including a low-threshold program via new media such as Facebook or Twitter could be beneficial. It could also be useful to assess impulsivity pre-operatively, though Sheets and colleagues [25] argue that especially post-operative variables seem to have an impact on weight loss outcome after bariatric surgery. Brandão and colleagues [27] hypothesized that impulsivity might be more associated with weight regain after surgery and not with weight loss. Thus, several post-operative measurement points concerning weight regain and weight loss outcomes should be completed. Another important point is that we did not especially include binge eating pathology in our analyses, though impulsivity represents a risk factor for BED [12,16] and binge eating has an impact on weight loss after bariatric surgery [28]. Subsequent studies should at best differentiate between patients after bariatric surgery with and without BED, as recommended by Schag and colleagues [16].

In conclusion, this study has contributed to elucidate the relationship between impulsivity and weight loss after bariatric surgery. According to our results, impulsivity has an indirect impact on weight loss through pathological eating behavior and mediated by depressive symptoms. Therefore, we think that the underlying factors of pathological eating behavior such as impulsivity and depression have to be regarded in the post-operative treatment of bariatric surgery patients.

## Figures and Tables

**Table 1:** Characteristics of patients four years after laparascopic sleeve gastrectomy.

	N	Mean	SD	Range
sex ^1^	65	39 ♀ 26 ♂	-	-
age (years)	65	49.0	±11.6	28.0–74.0
BMI (kg/m^2^)	64	36.9	±8.5	22.9–63.7
%EWL ^2^	64	52.0	±27.3	−20.7–113.3
%BWL ^2^	64	24.3	±12.4	−8.6–51.3
BIS-15 total score	59	30.4	±6.3	19–45
EDI-2 total score	54	239.1	±53.8	149–370
PHQ-9 score	61	6.7	±5.8	0–27

^1^ The frequency of male (♂) and female (♀) subjects instead of the mean is reported; ^2^ Positive values indicate weight loss. BIS-15: Barrat Impulsiveness Scale short version; BMI: body mass index; EDI-2: Eating Disorder Inventory version 2; %EWL: percentage excess weight loss; %BWL: percentage body weight loss; PHQ-9: Patient Health Questionnaire module depression; SD: standard deviation.

**Table 2:** Correlations among weight loss, impulsivity, depression, and pathological eating behavior.

	%EWL ^1^	BIS-15 Total Score ^1^	PHQ-9 Score ^2^	EDI-2 Total Score ^1^
%BWL ^1^	0.94 *	−0.16	−0.19	−0.41 *
%EWL ^1^	-	−0.16	−0.20	−0.39 *
BIS-15 total score ^1^	-	-	0.36 *	0.41 *
PHQ-9 score ^2^	-	-	-	0.72 *

* *p* < 0.001, two-sided. ^1^ Pearson correlation coefficient; ^2^ Spearman correlation coefficient. BIS-15: Barrat Impulsiveness Scale short version; EDI-2: Eating Disorder Inventory version 2; %EWL: percentage excess weight loss; %BWL: percentage body weight loss; PHQ-9: Patient Health Questionnaire module depression.
